# Biochemical and pathophysiological improvements in rats with thioacetamide induced-hepatocellular carcinoma using aspirin plus vitamin C

**DOI:** 10.1186/s12885-023-10644-5

**Published:** 2023-02-21

**Authors:** Rana R. El Sadda, Zahraa R. Elshahawy, Entsar A. Saad

**Affiliations:** 1 Chemistry Department, Faculty of Science, Damietta University, Damietta, 34517 Egypt; 2 Gastroenterology Surgical Center, Faculty of Medicine, Mansoura University, Mansoura, Egypt

**Keywords:** Caspase, Synergistic, CA19.9, AFP, BAX, p53, TNF-α

## Abstract

**Background:**

Hepatocellular carcinoma (HCC) is one of the leading causes of cancer-related death, so we should be concerned and look for effective/less-harmful treatments than chemotherapeutics already clinically in application. Aspirin works well ''in conjunction'' with other therapies for HCC since aspirin can boost the sensitivity of anti-cancer activity. Vitamin C also was shown to have antitumor effects. In this study, we examined the anti-HCC activities of synergistic combination (aspirin and vitamin C) vs. doxorubicin on HCC-bearing rats and hepatocellular carcinoma (HepG-2) cells.

**Methods:**

In vitro, we evaluated IC_50_ and selectivity index (SI) using HepG-2 and human lung fibroblast (WI-38) cell lines. In vivo, four rat groups were used: Normal, HCC (intraperitoneally (i.p.) administered 200 mg thioacetamide/kg/twice a week), HCC + DOXO (HCC-bearing rats i.p. administered 0.72 mg doxorubicin (DOXO)/rat/once a week), and HCC + Aspirin + Vit. C (i.p. administered vitamin C (Vit. C) 4 g/kg/day after day concomitant with aspirin 60 mg/kg/orally day after day). We evaluated biochemical factors [aminotransferases (ALT and AST), albumin, and bilirubin (TBIL) spectrophotometrically, caspase 8 (CASP8), p53, Bcl2 associated X protein (BAX), caspase 3 (CASP3), alpha-fetoprotein (AFP), cancer antigen 19.9 (CA19.9), tumor necrosis factor-alpha (TNF-α), and interleukin-6 (IL-6) using ELISA], and liver histopathologically.

**Results:**

HCC induction was accompanied by significant time-dependent elevations in all measured biochemical parameters except the p53 level significantly declined. Liver tissue architecture organization appeared disturbed with cellular infiltration, trabeculae, fibrosis, and neovascularization. Following drug medication, all biochemical levels significantly reversed toward normal, with fewer signs of carcinogenicity in liver tissues. Compared to doxorubicin, aspirin & vitamin C therapy ameliorations were more appreciated. In vitro, combination therapy (aspirin & vitamin C) exhibited potent cytotoxicity (HepG-2 IC_50_ of 17.41 ± 1.4 µg/mL) and more excellent safety with a SI of 3.663.

**Conclusions:**

Based on our results, aspirin plus vitamin C can be considered reliable, accessible, and efficient synergistic anti-HCC medication.

## Introduction

So far, among human diseases, cancer is the utmost mortal disease despite the prominent progression in therapies [1], with higher deaths registered in Developmental Countries [2]. The fifth most prevalent cancer in the world and the third lethal cancer overall is liver cancer [3–5]. Liver cancer was responsible for 7.69% of the universal cancer-linked deaths in 2020 [6]. It is the second-leading cause of cancer-related mortality among males and the fourth most common malignancy among them. Compared to women, men have a higher risk of developing liver cancer [7]. In terms of population, Egypt is the third and fifteenth most populated nation in Africa and the world, respectively. In Egypt, liver cancer is the fourth most prevalent cancer; it is the second among males and sixth among females [8]. In 2020, estimated cancer-linked deaths in Egypt were around 66% of the entire cancer-infected Egyptians [9]. Regarding primary liver cancer kinds, hepatocellular carcinoma (HCC) and intrahepatic cholangiocarcinoma (ICC) are the two main ones, while angiosarcoma, hemangiosarcoma, and hepatoblastoma are the less common ones. HCC develops from hepatocytes (primary liver parenchymal cells), whereas ICC begins in cholangiocytes (biliary cells) [10]. More than 90% of primary liver cancer cases worldwide are of HCC type [11]. Secondary liver cancer occurs when a tumor spreads to the liver from another body organ. A variety of malignancies like breast, lung, kidney, etc. can metastasize to the liver. However, secondary liver tumors majority come from colorectal cancer since about 70% of colon cancer patients seem to acquire subsequent liver cancer [12]. On the other hand, HCC risk markedly increases with hepatitis C virus (HCV) infection [13]. Cases of HCV or/and Hepatitis B virus (HBV) can progress to HCC [13, 14]. HBV/HCV co-infection can occur in areas with a high prevalence of these illnesses, like Egypt, increasing the risk of developing HCC by 20-fold [8, 15].

Clinically applied cancer chemotherapeutics cost high and suffer serious side effects like myelosuppression, anemia, and nephrotoxicity. Consequently, the innovation of novel, effective, and safe alternatives becomes mandatory [16]. For > 30 years, combined with other antitumor drugs, with surgery or radiation, the chemotherapeutic drug doxorubicin has been used as the first line of defense to cure many cancers [14, 17]. Doxorubicin is hazardous to many organs, particularly the heart, so if necessary, it must be used with precaution while treating cancer [14]. Yet doxorubicin-associated worse side effects like cardio-, neuro-, and nephrotoxicity are significant obstacles facing clinical application. Doxorubicin load and the patient's qualification of bone marrow for renewal specify the seriousness of these side effects and how frequently they happen [18].

Aspirin (acetylsalicylic acid) has been known as an antipyretic for over two hundred years. During Hippocrates, aspirin was used as an analgesic. Aspirin was first made available in the late 1890s. It has been employed to treat many inflammatory diseases [19]. Aspirin, too, has been discovered to reduce the risk of developing some malignancies by frustrating the cyclooxygenase enzyme [20]. Gu et al., 2005, reported that aspirin causes apoptosis in gastric cancer via changing Bcl2 associated X protein (BAX) conformation, moving BAX to the mitochondria, and activating caspase 8 (CASP8)/BH3 Interacting Domain Death Agonist (Bid) and caspase 3 (CASP3) [21]. Aspirin has proven to be a cancer preventive by clinical investigations. According to numerous studies, aspirin appears to have a positive effect on HCC when used as a chemo-preventive or adjuvant chemotherapeutic drug. Moreover, aspirin's anti-platelet and anti-inflammatory properties might work as an adjuvant to other treatments to lessen the return of HCC [22].

In addition to being an antioxidant and a possible cofactor for a variety of biosynthetic and gene regulatory proteins, vitamin C (L-ascorbic acid or ascorbate) is a crucial micronutrient for humans. Vitamin C supports immunological defense by assisting numerous cellular processes of innate and adaptive immune systems. Vitamin C can boost chemotaxis, phagocytosis, the production of free radicals, and eventually microbial death by ingathering in phagocytic cells like neutrophils [23]. Vitamin C may have antitumor effects; some research papers have shown that vitamin C can induce anticancer activity in various cell lines [24]. Moreover, in 2018, Lv et al. introduced vitamin C as an effective eliminator of liver cancer stem cells (CSCs) and a killer of cancer cells in the liver [25].

Therefore, and because safe and selective drugs should always be a concern, the main goal of our study is to investigate using a synergistic combination of aspirin & vitamin C as an alternative for doxorubicin in the hope it would be efficient and safer in treating liver cancer.

## Materials and methods

### In vitro study

#### Cell lines

Human lung fibroblast (WI-38) and Hepatocellular carcinoma (HepG-2) cell lines from the Holding company for biological products and vaccines (VACSERA), Cairo, Egypt, were used.

#### Chemical reagents

3-(4,5-Dimethylthiazol-2-yl)-2,5-diphenyltetrazolium bromide (MTT), Roswell Park Memorial Institute (RPMI)-1640 medium, and dimethyl sulfoxide (DMSO) were obtained from Sigma Co., St. Louis, USA, but Fetal Bovine serum (FBS) was supplied from GIBCO, UK.

#### MTT assay

To determine the inhibitory effects of compounds on cell growth of the two cell lines mentioned above, the MTT assay [26], as described in [27] and [28], was applied.

#### The selectivity index (SI)

The SI was calculated using the formula: SI = (IC_50_ for normal cell line WI-38)/(IC_50_ for cancerous cell line HepG-2).

### In vivo study

**Fig. 1:**
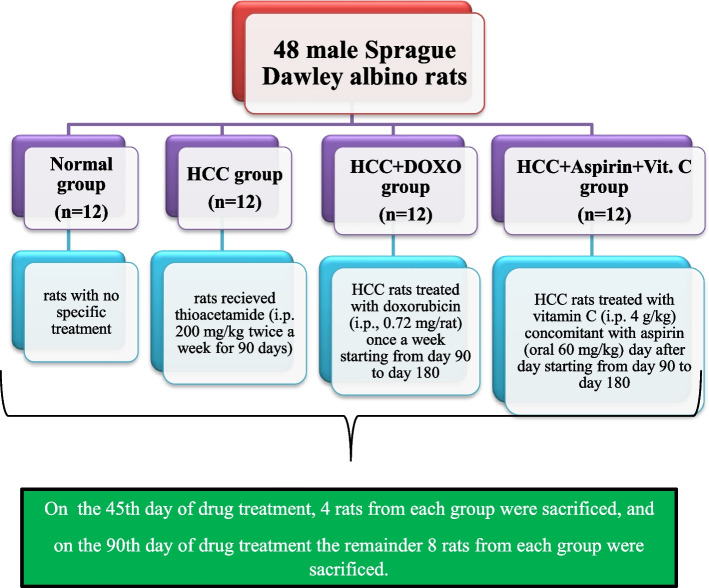
Schematic diagram for the experimental design

Forty-eight male Sprague Dawley albino rats (weight: 200–220 g and age: 8–10 weeks) were received from the National Research Center (NRC, Cairo, Egypt) and housed randomly, at five rats per cage. They received human care in compliance with the standards in the Guide for the Care and Use of Laboratory Animals published by the National Institute of Health, and approved by the Animal House of Biochemistry, Chemistry Department, Faculty of Science, Damietta University, Damietta, Egypt. Rats were kept at 23 ± 2 ºC, with controlled humidity, and 12/12 h light/dark cycle. They were fed on a normal laboratory rodent diet, and given water ad libitum. They were ''carefully'' observed daily. Their body weights were recorded, while food and water intakes were accurately measured each week to assess any signs of toxicity or abnormality throughout the experiment. Rats were divided into four groups (*n* = 12, Fig. 1) as follows:

- Group 1 (Normal): healthy rats with no specific treatment.
- Group 2 (HCC): rats intraperitoneally (i.p.) administrated thioacetamide (TAA) at a dose of 200 mg/kg/day [29] twice a week for 90 days.
- Group 3 (HCC + DOXO): HCC rats were treated i.p. with doxorubicin at a dose of 0.72 mg/rat/day which is equivalent to the human dose (20 mg/m^2^) [30], once a week starting from day 90 to day 180.
- Group 4 (HCC + Aspirin + Vit. C): HCC rats were treated i.p. with vitamin C (Vit. C) day after day at a dose of 4 g/kg/day [31] concomitant with aspirin day after day using oral gavage at a dose of 60 mg/kg/day [32] starting from day 90 to day 180.

For investigation of the drug treatment duration effect, we killed four animals from each group on the 45th day of drug treatment and the remainder of eight animals on the 90th day of drug treatment. Blood samples, via penetration of retro‐orbital bleeding plexus, and livers were collected from sacrificed rats under combined ketamine (75 mg/kg)/xylazine (10 mg/kg) anesthesia. Livers were rinsed with saline, dried, weighed, photographed, thereafter fixed in 10% formalin for usage in the histopathology study. Sera were routinely separated from the blood samples after clotting and saved at -20 ºC until needed.

### Biochemical investigations

Liver function tests were estimated by applying instructions of the manufacturer for albumin, aspartate aminotransferase (AST), and alanine aminotransferase (ALT) kits from Biomerieux, 100 Rodolphe Street, Durham, USA, while for total bilirubin (TBIL) kit from Roche Diagnostic, Sadholer Street 116, Mannheim, Germany. Tumor markers (alpha-fetoprotein (AFP) and cancer antigen 19.9 (CA19.9)) concentrations were determined according to kits from Roche Diagnostic, Sadholer Street 116, Mannheim, Germany. Assays of interleukin-6 (IL-6), BAX, and CASP3 were done using Enzyme-linked Immunosorbent Assay (ELISA) kits from Cloud-crone corp., Unit 2201, Katy, TX, USA. Regarding tumor necrosis factor-alpha (TNF-α) level, it was estimated by applying Rat Tumor Necrosis Factor Alpha (TNFA) ELISA kit from Abbexa, Cambridge Science Park, Cambridge, UK. Estimation of CASP8 was done via ELISA for quantitative detection of Rat CASP8 from Novus Biological, Briarwood Avenue, USA. Finally, the p53 level was estimated using the sandwich enzyme immunoassay kit from Cusabio kit, Fannin Street, Huston, TX, USA.

### Histopathological analysis

Samples of livers fixed in 10% formalin for the routine hematoxylin and eosin (H&E) staining technique and histopathological examinations were processed routinely, embedded in paraffin wax, cut into five µm thick sections in a rotary microtome, and stained with H&E dye. Three-different sections, at least, were examined per one liver sample.

### Statistical analysis

Data were analyzed using the Statistical Package for the Social Sciences [33] version 17 (SPSS Inc. Released 2008. SPSS Statistics for Windows, Version 17.0. Chicago, USA) and expressed as mean ± standard error (SE). For data with Gaussian distribution, statistical analysis was performed using analysis of variance (One Way ANOVA) followed by the Bonferroni multiple comparisons test. For parameters with non‑Gaussian distribution, Kruskal–Wallis test was employed, followed by Dunnett’s test for multiple comparisons. Differences were considered significant at *p* < 0.05.

## Results

### Cytotoxicity effect of aspirin and vitamin C combination

**Fig. 2:**
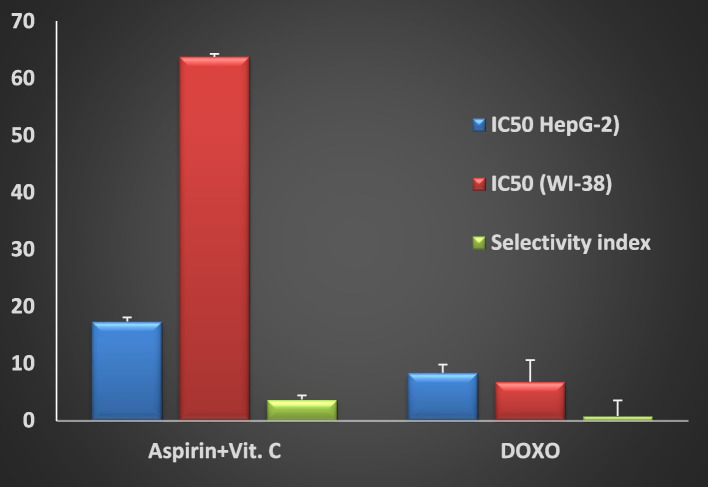
The drug concentration (µg/mL) to inhibit cell proliferation by 50% (IC_50_) of each cell line and the selectivity index. IC_50_: 1–10 is very strong, 11–20 is strong, 21–50 is moderate, 51–100 is weak, and > 100 is non-cytotoxic [34]

Figure 2 shows that the combination of aspirin and vitamin C is a strong-cytotoxic for human liver cancer cell line (HepG-2, IC_50_ = 17.41) and is a weak toxic (safe) for healthy cells derived from lung tissue (WI-38, IC_50_ = 63.79). On the other hand, doxorubicin is very cytotoxic for both cancer (HepG-2) and healthy cells (WI-38); IC_50_ of 8.35 and 6.84 µg/mL, respectively. The selectivity index (SI) for ‘’aspirin & vitamin C’’ and doxorubicin equals 3.663 and 0.819, respectively. The higher SI, the higher selection for cancer cells and the safer for healthy cells.

### Surface changes of excised livers

**Fig. 3:**
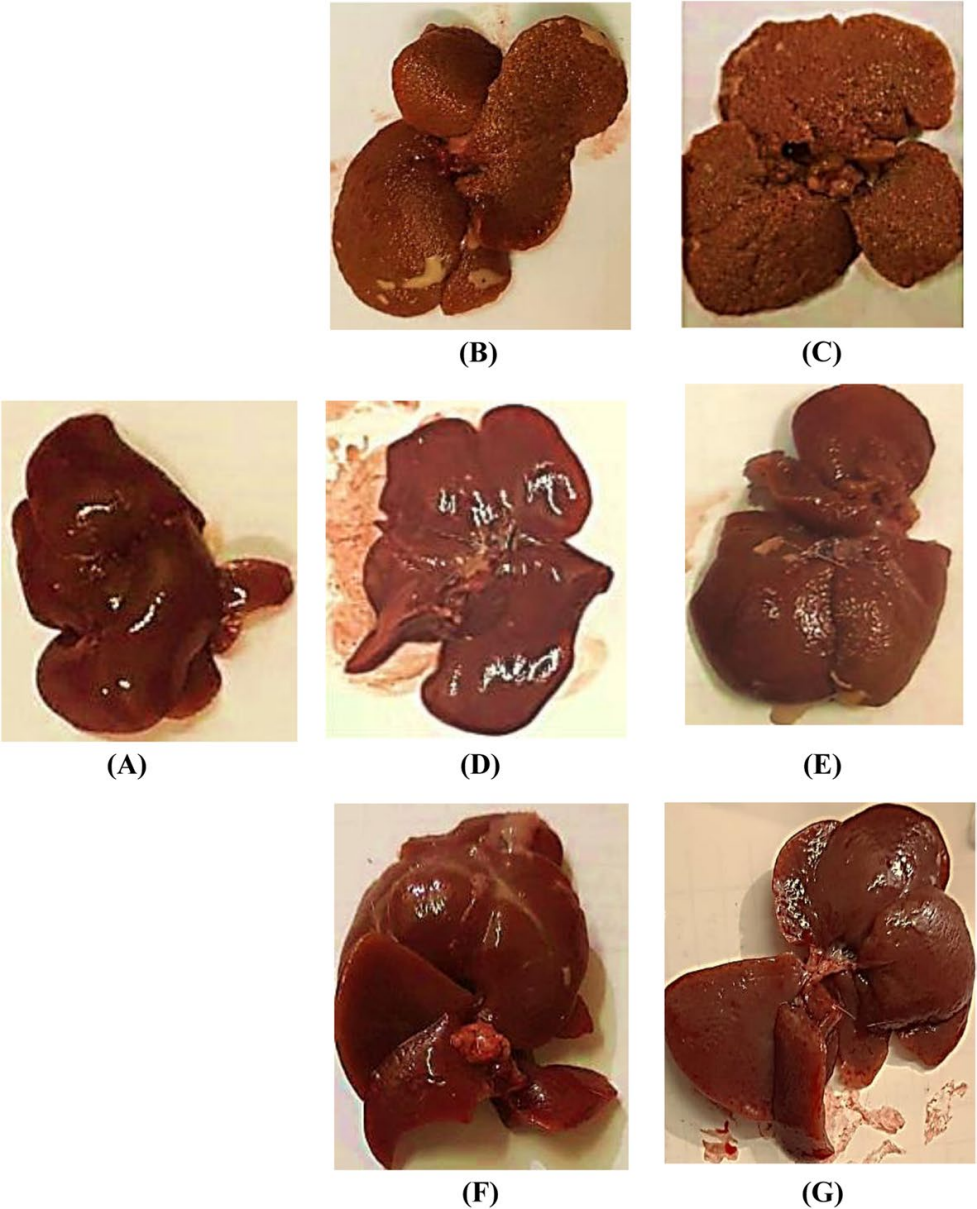
Excised liver representative photos. **A** Normal, **B** HCC after 45 days of thioacetamide (TAA) injection, **C** HCC after 90 days of TAA injection, **D** HCC after 45 days of DOXO treatment, **E** HCC after 45 days of Aspirin + Vit. C treatment, **F** HCC after 90 days of DOXO treatment, **G** HCC after 90 days of Aspirin + Vit. C treatment

HCC rats had irregular hepatic surfaces (several small nodules and granular appearance) and faint color compared with the normal ones. The livers were restored towards healthy smooth morphologies after treatment of HCC rats with doxorubicin or ''aspirin & vitamin C'' with differences according to treatment duration for 45 or 90 days; some of those treated for 45 days showed little scars; it will take time to heal damaged liver tissues completely. Treatment with aspirin & vitamin C was the best in restoring healthy liver morphology. Besides the duration of treatment, it is another factor that must be concerned (Fig. 3).

### Biochemical markers

**Fig. 4:**
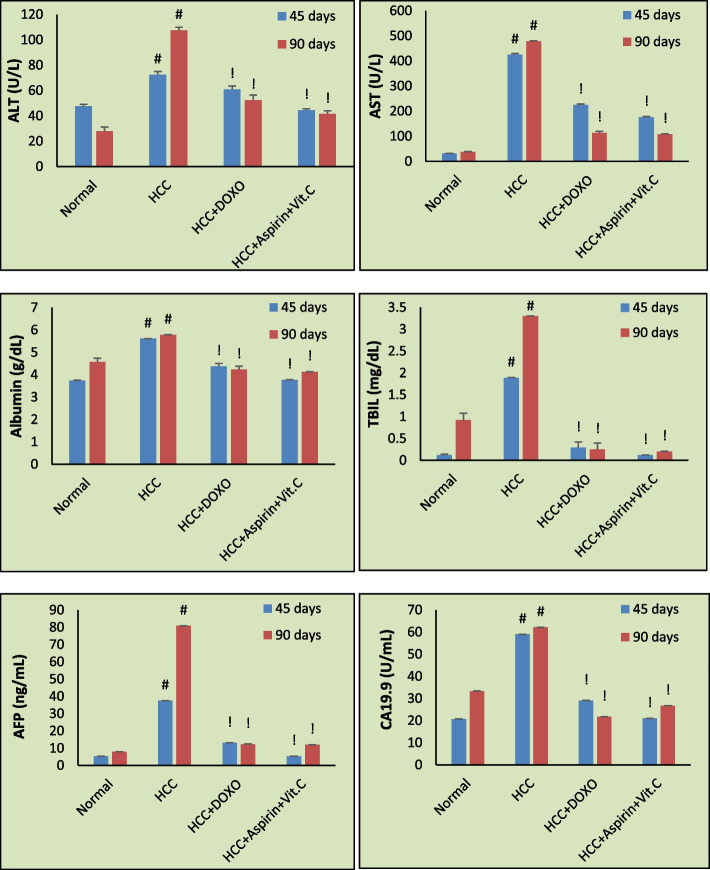
Mean serum levels of alanine aminotransferase (ALT), aspartate aminotransferase (AST), albumin, total bilirubin (TBIL), alpha-fetoprotein (AFP), and cancer antigen 19.9 (CA19.9) in different groups at 45 days and 90 days of treatment. Data are expressed as mean ± SE. #: *p*˂0.001 against Normal group. !: *p*˂0.001 against HCC group

**Fig. 5:**
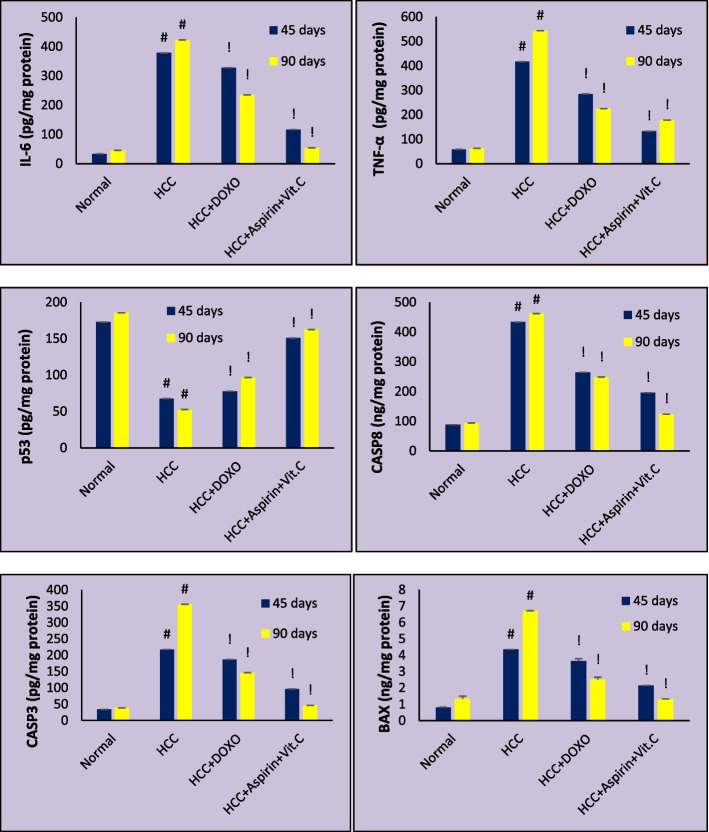
Mean serum levels of interleukin-6 (IL-6), tumor necrosis factor-alpha (TNF-α), protein 53 (p53), caspase 8 (CASP8), caspase 3 (CASP3), and Bcl-2-associated x protein (BAX) in different groups at 45 days and 90 days of treatment. Data are expressed as mean ± SE. #: *p*˂0.001 against Normal group. !: *p*˂0.001 against HCC group

For the HCC model, injection of carcinogenic TAA caused increases in ALT, AST, albumin, TBIL, AFP, CA19.9, IL-6, CASP3, CASP8, and BAX levels significantly compared with normal rats. p53 level was significantly decreased compared with normal rats. The above changes were more pronounced with time, as shown for 45 and 90 days (Figs. 4 and 5).

After HCC treatment by the commonly used chemotherapy drug (doxorubicin) or ''aspirin & vit. C'' we noticed a significant increase in p53 level and significant decreases in ALT, AST, albumin, TBIL, AFP, and CA19.9, IL-6, CASP3, CASP8, and BAX levels compared to HCC rats as in Figs. 4 and 5. The best improvements were associated with rats treated with aspirin and vitamin C, not doxorubicin. We also noticed that treatment improvements were duration-dependent.

### Histopathological study

**Fig. 6:**
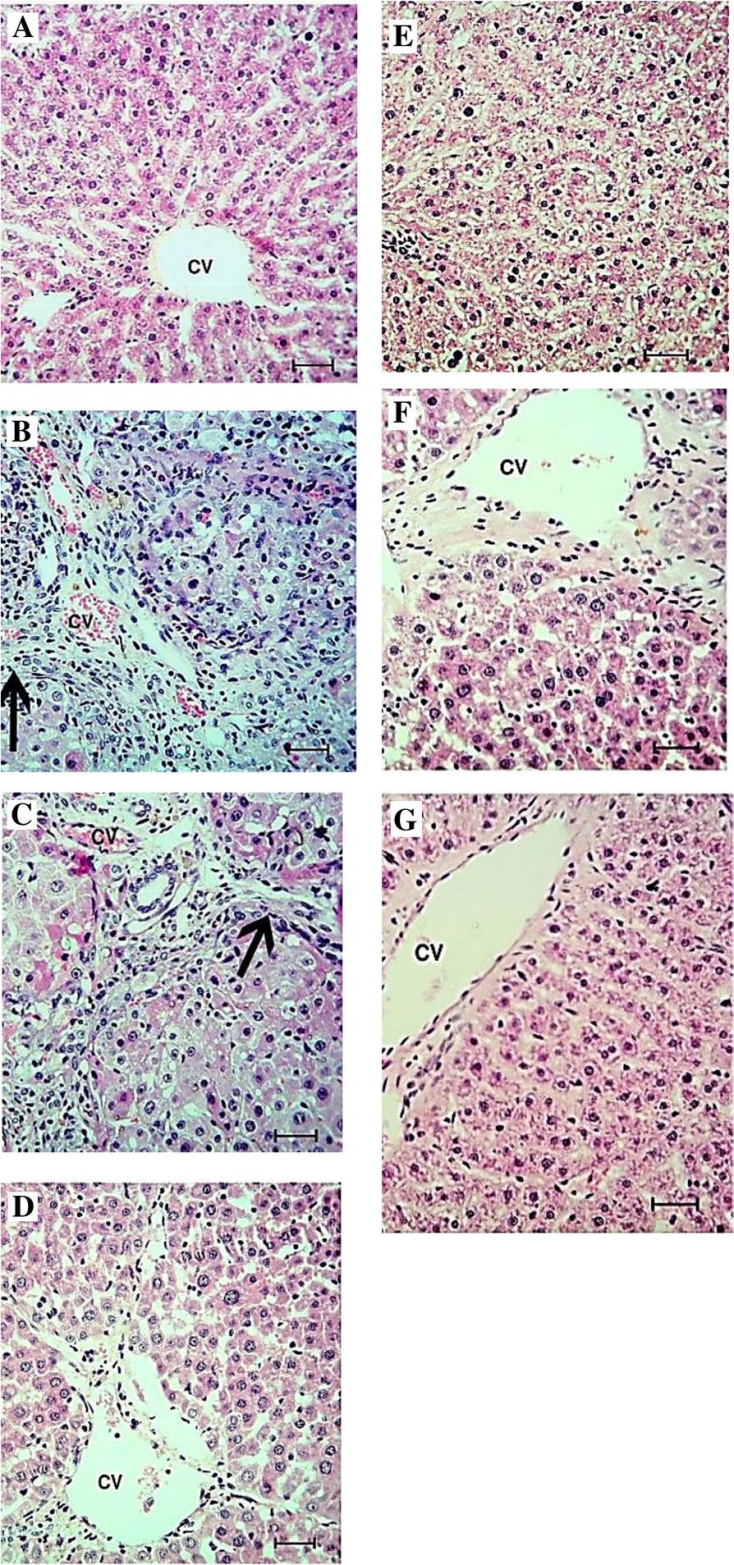
**A** shows the histological section of the Normal control liver of rats with the normal liver structures. **B** is the HCC at 45 days showing disruption of the hepatocytes organization with formation of pleomorphism, moderate cellular infiltrate (**+ +**) and pseuoacini separated by fibrous trabeculae (arrow). The individual hepatocytes show nonuniform eosinophilia of the cytoplasm with high N/C ratio. **C** is the HCC at day 90, the liver section appears more disturbed with denser trabeculae (arrow), mild fibrosis (** +**) and neovascularization. **D** & **E** shows HCC treated with Doxorubicin (DOXO) at 45 days (necrosis +) and 90 days (tumor formation and apoptosis +), respectively; signs of carcinogenicity are fewer. **F** & **G** shows the histological section of the HCC treated with the combination of Aspirin & Vit. C at 45 and 90 days, respectively; most of the liver tissue appears normal (X: 200 bar 50 µm). CV: central vein

Figure 6A showed normal healthy liver. On day 45 (Fig. 6B), HCC liver tissues showed pleomorphism, moderate cellular infiltrates (+ +), and pseudo-acini separated by fibrous trabeculae with hepatocytes showing a high N/C ratio. On day 90 (Fig. 6C), the HCC liver section appeared more disturbed with denser trabeculae, mild fibrosis ( +), and neovascularization. After HCC treatment with doxorubicin for 45 days (Fig. 6D), sections showed necrosis ( +), while for 90 days (Fig. 6E), tissues showed tumor formation and apoptosis ( +). On the other side, the liver sections of treated HCC rats with ''aspirin & Vit. C'' for 45 and 90 days (Fig. 6F & G) showed almost normal liver histology.

## Discussion

In this study, the cytotoxicity test of drugs using the MTT assay against both carcinoma cells HepG2 and healthy cells WI-38 was performed first to show effectiveness and safety. It showed IC_50_ for HepG-2 and WI-38 of 17.41 ± 1.4 and 63.79 ± 3.8 µg/mL, respectively, for ''aspirin and vitamin C'' vs. 8.35 ± 0.7 and 6.84 ± 0.5 µg/mL for doxorubicin indicating that ''aspirin and vitamin C'' therapy is strong cytotoxic for cancer but safe for healthy cells. At the same time, doxorubicin (the reference chemotherapy) is very strong toxic to cancer cells but still very strong cytotoxic to normal cells. The SI evaluates the toxicity of the compounds against normal cells and predicts their therapeutic potential. The big difference between cytotoxicity versus cancer and normal cells is the higher SI values. High SI values imply malignant cells will be killed at a higher rate than normal cells [35]. Hence, the calculated SI ratio of 3.663 and 0.819 for ''aspirin and vitamin C'' and doxorubicin, respectively, is further evidence indicating that applying ''aspirin and vitamin C'', contrary to doxorubicin, for treating HCC is expected to show minimal/or no toxicity for healthy cells. Another study by Tsakalozou et al. reported that doxorubicin has a high cytotoxic effect; they used docetaxel in combination with doxorubicin to treat prostate cancer cell line PC3 to reduce doxorubicin's high cytotoxicity effect on normal cells [36].

Liver morphological and histopathological examination introduced clear evidence for HCC development and time-dependent progression. The malignant livers were granular with dispersed nodules and paled color, as clearly seen in Fig. 3B&C. HCC liver sections (Fig. 6B&C) showed disturbed organization of cells, dense trabeculae, and neovascularization. In our TAA-induced HCC model, elevated biochemical tumor markers, AFP and CA19.9 are other evident signs of HCC development. Besides, high TBIL, increased AST, and a lesser increase in ALT levels are indications of liver dysfunction. Routinely, the chief common diagnostic sign of liver chemistry deterioration is high levels of aminotransferases [37] as a result of cellular infiltration, evidenced histologically here in Fig. 6B, with AST levels, more frequently, higher than ALT levels due to decreased AST clearance or/and flow of mitochondrial AST linked with severe liver architectural destruction [1, 38]. Rapid red blood cells destruction due to shortened lifespan owed to oxidative damage-red cell membrane destruction in addition to spreading of HCC cancer that may close the bile duct or distort the performance of the liver's typical functioning rise TBIL [39]. Compared to doxorubicin, the 90-day treatment of HCC using ''aspirin & vitamin C'' was shown to highly improve the liver's morphology, as seen in Fig. 3G, and the functional state as well as the hepatic architecture, where most of the liver tissue seen in Fig. 6G appeared normal, and reduce the leakage of hepatocytes injury markers (ALT, AST, and TBIL) into the circulation. For complete recovery, longer treatment duration may be necessary. We recorded a decrease in albumin in treated HCC groups (*p* < 0.001) compared with untreated HCC rats. By contrast, malignant hepatocytes of HCC-bearing rats showed more albumin production because they have a lesser ability to control albumin synthesis or because of HCC-linked renal dysfunction. Our findings are compatible with El-Emshaty et al. [13], Ozaslan et al. [40], and Raju and Arockiasamy [41]. They demonstrated disturbed liver functions in cancer-bearing groups. Seaton and his team also noted that albumin stabilizes deoxyribonucleic acid (DNA) replication and cell proliferation, which explains why albumin content rose in HCC patients [42].

In the current work, the high rise in inflammation markers levels TNF-α and IL-6 in HCC-bearing rats ‘’indirectly’’ suggest that hepatocytes are under severe oxidative stress supported obviously by cellular infiltrate seen in liver sections of untreated HCC rats (Fig. 6B). In that way, TAA poison administration results somehow in the loss of some healthy liver tissue at the expense of cancer formation and progression. That loss induces inflammation as a logical response to dispose of tissue wreckages prior to the regenerative response. This hypothesis is in harmony with Carnovale et al. [43]. Thus, cancer can take advantage of healthy cells' tools and events to provide an optimum environment for cancerous cells to grow and expand. Our proposed scenario is as follows: TAA-derived free radicals release contributes to oxidative stress, and under oxidative stress, TNF-α is inspired to bind TNF receptor 1 (TNFR1) resulting in the turning of CASP8 into its active form [44]. Active CASP8 can (1) in the intrinsic pathway of apoptosis: convert Bid to truncated Bid (tBid) that get enter mitochondria, and encourages cytochrome C flow-out then both caspase 9 (CASP9) & cytochrome C with other factors give apoptosome and activates CASP3 leading ultimately to cellular death, or (2) in the extrinsic pathway of apoptosis: activate CASP3 starting cellular death events [28]. Besides, severe oxidative stress could elevate BAX levels that encourage cytochrome C release, and apoptotic events go ahead. Based on all, a wave of healthy hepatocyte death exists, evidenced through our findings from rises in apoptotic CASP8 & CASP3 and pro-apoptotic BAX levels in the HCC group. On the other side, the noticed high decrease in p53 level in the HCC group mostly favors the survival of cancerous cells and encourages cancer mass expansion [45], as evidenced by our histopathological findings concerning untreated HCC liver sections; hepatocytes were of disturbed organization with dense trabeculae, they showed pleomorphism, nonuniform eosinophilia of the cytoplasm with high N/C ratio, infiltrates, fibrosis, and neovascularization (Fig. 6B). All these manifestations were gotten worse with the longer duration of TAA administration (90 days, Fig. 6C). HCC-bearing rats kept on the administration of ''Aspirin & vitamin C''/or doxorubicin drug showed reversal of these inflammation and apoptotic markers concentrations towards near normal ranges indicating HCC collapse and restoration of both liver architecture and function to their near normal status. Aspirin & vitamin C medication for 90-days showed the most positive impact compared to doxorubicin.

Future studies to investigate the extended effects of the treatment on organs other than the infected liver are still needed. Further studies on different animal models and with a longer duration of vitamin C & aspirin treatment are also required to validate our outcomes.

## Conclusions

According to our research, synergistic vitamin C & aspirin treatment of HCC rats is superior to doxorubicin treatment. When compared to each other via cytotoxicity test and selectivity index, ‘’vitamin C & aspirin’’ are safe and selective. On the other hand, aspirin and vitamin C work well as anti-inflammatory, anti-apoptotic, and antitumor treatments. One should always consider the length of the course of treatment. Its increase from 45 to 90 days was optimal for regaining a near normal and healthy liver. Up on our results, we advise taking into account aspirin & vitamin C as a synergistic anti-HCC medicine that is safe, readily available, and effective.

## Data Availability

The datasets used and analyzed during the current study are available from the corresponding author on reasonable request.
